# Molecular, phylogenetic and developmental analyses of Sall proteins in bilaterians

**DOI:** 10.1186/s13227-018-0096-z

**Published:** 2018-04-10

**Authors:** José Lorente-Sorolla, Marta Truchado-Garcia, Kimberly J. Perry, Jonathan Q. Henry, Cristina Grande

**Affiliations:** 1 Departamento de Biología Molecular and Centro de Biología Molecular “Severo Ochoa”, Consejo Superior de Investigaciones Científicas, Universidad Autónoma de Madrid, Madrid, Spain; 2 Present Address: Departamento de Biología, Facultad de Ciencias, Universidad Autónoma de Madrid, Madrid, Spain; 3 Department of Cell and Developmental Biology, University of Illinois, 601 S. Goodwin Avenue, Urbana, IL 61801 USA; 4 Departamento de Biología, Facultad de Ciencias, Universidad Autónoma de Madrid, C/Darwin, 1; Cantoblanco, 28049 Madrid, Spain

**Keywords:** *Lottia gigantea*, *Crepidula fornicata*, Gastropoda, Spiralia, Gene evolution, Protein domains, Spalt, Sall

## Abstract

**Background:**

Sall (Spalt-like) proteins are zinc-finger transcription factors involved in a number of biological processes. They have only been studied in a few model organisms, such as *Drosophila melanogaster*, *Caenorhabditis elegans*, *Schmidtea mediterranea* and some vertebrates. Further taxon sampling is critical to understand the evolution and diversification of this protein and its functional roles in animals.

**Results:**

Using genome and transcriptome mining, we confirmed the presence of *sall* genes in a range of additional animal taxa, for which their presence had not yet been described. We show that *sall* genes are broadly conserved across the Bilateria, and likely appeared in the bilaterian stem lineage. Our analysis of the protein domains shows that the characteristic arrangement of the multiple zinc-finger domains is conserved in bilaterians and may represent the ancient arrangement of this family of transcription factors. We also show the existence of a previously unknown zinc-finger domain. In situ hybridization was used to describe the gene expression patterns in embryonic and larval stages in two species of snails: *Crepidula fornicata* and *Lottia gigantea*. In *L. gigantea*, *sall* presents maternal expression, although later on the expression is restricted to the A and B quadrants during gastrulation and larval stage. In *C. fornicata*, *sall* has no maternal expression and it is expressed mainly in the A, C and D quadrants during blastula stages and in an asymmetric fashion during the larval stage.

**Discussion:**

Our results suggest that the bilaterian common ancestor had a Sall protein with at least six zinc-finger domains. The evolution of Sall proteins in bilaterians might have occurred mostly as a result of the loss of protein domains and gene duplications leading to diversification. The new evidence complements previous studies in highlighting an important role of Sall proteins in bilaterian development. Our results show maternal expression of *sall* in the snail *L. gigantea*, but not *C. fornicata*. The asymmetric expression shown in the ectoderm of the trochophore larva of snails is probably related to shell/mantle development. The observed *sall* expression in cephalic tissue in snails and some other bilaterians suggests a possible ancestral role of *sall* in neural development in bilaterians.

**Electronic supplementary material:**

The online version of this article (10.1186/s13227-018-0096-z) contains supplementary material, which is available to authorized users.

## Background

**Fig. 1:**
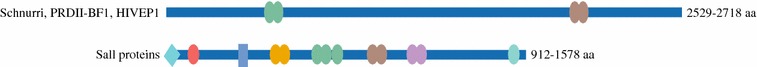
Organization of conserved domains in “Sal-box” containing proteins. Colored ovals represent the zinc-finger motifs. The blue rectangle represents the poly-Q region. The turquoise diamond represents the 12 conserved amino acids at the N-terminal end of the Sall proteins that interact with the HDC NuRD [14]. Protein length range is indicated at the right

Sall (Spalt-like) proteins are zinc-finger transcription factors that range from 105 to 140 kDa, and are characterized by the presence of several zinc-finger (ZF) domains distributed along the protein [1] and a glutamine-rich region (poly-Q) between the ZF1 and ZF2 [2] (Fig. 1). Four of these ZF domains are formed by zinc-finger motifs arranged in pairs connected by the evolutionarily conserved inter-finger “spacer” H/C link motif [3], and each C-terminal zinc finger of the pair contains the “Sal-box” (FTTKGNLK), also present in other zinc-finger proteins, such as Schnurri [4, 5], HIVEP1 and PRDII-BF1 [6] (Fig. 1).

The *spalt* (*sal*) genes were originally described as homeotic genes in *Drosophila melanogaster* [2], which has two paralogs: *spalt major* (*salm*) and *spalt-related* (*salr*) [7]. Afterward, *spalt* orthologs have been also described in the nematode *Caenorhabditis elegans* [8], the planarian *Schmidtea mediterranea* [9], as well as in some species of vertebrates [10]. All these studies have shown that *spalt* is activated in response to several signal transduction pathways in different tissues and developmental processes [11], and the expression patterns and functions of *sall* genes, together with the analysis of their regulation, indicate they cannot be universally assigned to a specific signaling pathway.

For instance, Sall proteins have been described as transcriptional repressors, mainly through two mechanisms [12]: (1) the interaction between 12 amino acids located at the N-terminal part of Sall proteins and the histone deacetylase complex NuRD [13–15] and (2) the direct binding to an AT-rich region of the heterochromatin of the central region of Sall proteins that includes ZF2 and ZF3 pairs [16]. In addition, Sal1 proteins can interact with PIN2, an isoform of telomeric repeat-binding factor 1 (TRF1), which might indicate an involvement of Sall proteins in the regulation of higher-order chromatin structures and that the Sall proteins could be components of a distinct heterochromatin-dependent silencing process [17]. In addition, Sall proteins have also been described as transcriptional activators of several genes, such as the cyclin CDK inhibitor *p21* [18], *Nanog* [19], *Pou5f1* [20] and *Sall* itself [21].

The subcellular localization and transcriptional capacity of Sall proteins might be conditioned by posttranslational modifications and protein interactions. SUMOylation modifies its localization inside the nucleus [12], while phosphorylation reduces its repression activity [22]. Interaction between Sall proteins can also have functional consequences, such as differences in subcellular localization that might be mediated by the poly-Q region [23].

Sall is therefore involved in a number of different biological processes. The *Drosophila sal* homeotic genes are implicated in many developmental processes [11, 12] such as the specification of head and tail during embryogenesis [24], organogenesis [25–27] and the determination of neural fate in the peripheral nervous system [28–30]. Similarly, in the nematode *C. elegans*, the *sall* gene *sem*-*4* controls the fate of several different cell types including neurons, muscle, hypodermis, sex myoblasts, coelomocytes and multiple neuronal lineages [8, 31]. Studies in the nematode and also in flies and vertebrates indicate that *sall* genes might regulate this patterning and cellular identity through repression of Hox genes [32]. In addition, *sal* is involved in the formation of excretory systems in planarians [9]. Finally, vertebrate homologs of *spalt* (SALL) have been shown to be involved in normal development and tumor suppression and are implicated in several human genetic disorders [10]. They have important roles during neural development [20, 33–35] and organogenesis, especially in kidney [9, 34, 36–38], heart [33, 34, 36] and limb development [23, 39, 40].

Therefore, knowledge about *spalt* and its expression derives from studies in a few model organisms representing the three major clades of bilaterians: Ecdysozoa *D. melanogaster* Salm and Salr and *C. elegans* SEM-4; Spiralia *S. mediterranea* Sall; and Deuterostomia Vertebrate Sall proteins. Unlike the deuterostomes and ecdysozoans, spiralians have not been so widely used in modern cell and molecular research. Spiralians comprise 14 morphologically diverse phyla (including mollusks, annelids and nemerteans) that share some developmental processes like the presence of spiral cleavage, from which the name of the group is derived [41]. The diversity of developmental programs, life histories and body plans makes them an excellent group to undertake comparative studies aimed at understanding the molecular and genetic bases of the evolution of morphological diversity of triploblast bilaterian metazoans.

Advances in sequencing have made available new genomic and transcriptomic data for some non-model Spiralia. The snail species *Lottia gigantea* was the first representative of Spiralia to have its genome sequenced [42], and the availability of sequence data for other members of this group in public databases like NCBI keeps increasing by the day. In addition, more studies have recently focused on the developmental genetics of Spiralia in the last few years, mostly with a focus on understanding the role of certain genes in the origin of animal body plan diversity and in molecular evolution of genes and genetic pathways. Although there are no classical model species in developmental genetics of Spiralia, some efforts of several labs including ours have focused on a few species of snails, nemerteans and annelids, which have provided valuable information on gene expression patterns and on experimental manipulation of certain genes [43–48]. One conserved feature shared by the spiralians is the distinct pattern of alternating oblique embryonic cell divisions referred to as “spiral cleavage.” In spiralian embryos, the first and second orthogonal cleavage planes led to the formation of four cells (macromeres A, B, C and D) and the progeny of these cells will define the future left, ventral, right and dorsal body sides (referred to as A, B, C and D “quadrants,” respectively). Subsequent divisions result in the stereotypic formation of successive tiers of generally smaller micromeres, which are generated toward the animal pole. Cleavage is then followed by gastrulation and finally by the formation of a larva [49].

In order to understand the origin and the evolutionary history of Sall proteins in bilaterians, here we have identified Sall proteins in several Spiralia, including mollusks, an annelid and a brachiopod, as well as in some other bilateral organisms. We have identified the main protein domains and the potential protein interaction sites in all these newly determined Sall orthologs. We also report the expression pattern of *sall* gene in two species of gastropods (*L. gigantea* and *Crepidula fornicata*) during their embryonic development. Finally, we discuss the evolution of Sall proteins, the evolutionary conservation of their domains, and the temporal and local activation in a wider comparative phylogenetic analysis.

## Methods

### Identification of candidate Sall proteins

In order to identify candidate Sall proteins, we performed searches in different databases (Additional file 1: Table S1). Potential snail Sall sequences were derived from *Biomphalaria glabrata* and *C. fornicata* from RNA-seq datasets generated in our lab [50] and uploaded to Geneious version 6.1.2 [51]. Potential sequences of the snail *L. gigantea* were retrieved from the JGI genome portal [42] using tblastn and pblast alignment algorithms [52]. Potential *sall* orthologs for the Xenoturbellid *Xenoturbella*, the acoels *Convolutriloba* and *Isodiametra*, the annelid *Dinophilus*, the brachiopods *Terebratalia* and *Novocrania*, the nemertean *Lineus*, the priapulid *Priapulus*, the platyhelminth *Prostheceraeus*, the nemertodermatid *Meara*, and the bryozoan *Membranipora* were searched for in RNA-seq datasets (Additional file 1: Table S1). In order to have more representatives of other bilaterian (arthropods, nematodes, mollusks, tunicates, echinoderms, hemichordates, vertebrates) and non-bilaterian clades (cnidarians, ctenophorans, placozoans, poriferans) for a wider analysis of the phylogeny and structure of Sall proteins, additional searches were performed in the NCBI databases [53] using keyword search (Spalt, Spalt-like, sall, sal-like), tblastn and pblast. In addition, the zinc-finger proteins containing the Sal-box motif Schnurri, PRDII-BF1 and HIVEP1 were also retrieved from the NCBI databases using keyword search (Schnurri) and tblastn and pblast search using the Sal-box motif as template. Translation into protein sequences was carried out using MacVector version 12.7 [54], assuming standard codon usage.

### Phylogenetic analyses

Full-length sequences of available Sal-box containing proteins (Schnurri, PRDII-BF1, HIVEP1) (Additional file 1: Table S1) were aligned with Sall potential orthologs (Additional file 2: Fig. S1) using ClustalX version 2.1 [55] followed by refinement by eye and trimmed in MacVector, selecting the homologous sequences and excluding sites of ambiguous alignment and gaps. In order to determine whether the newly determined potential Sall proteins were indeed Sall proteins or other proteins containing a Sal-box, we performed a phylogenetic analysis including the zinc-finger domains three and four (ZF3 and ZF4), the only two zinc-finger domains present in all proteins containing a Sal-box (Fig. 1; Additional file 2: Fig. S1), for all the sequences retrieved in this study. Once orthology was established, a second phylogenetic analysis was performed including the zinc-finger domains two, three and five (ZF2, ZF3 and ZF5) (Additional file 3: Fig. S2) for all the Sall sequences retrieved in this study (Additional file 1: Table S1). These two datasets were subjected to coalescent-based, Bayesian inference (BI) phylogenetic analyses implemented using BEAST 1.8.3 software [56]. The JTT + G model [57] was selected as the best-fit model of protein evolution using ProtTest [58]. We assumed a strict molecular clock and the Yule speciation model as the coalescent prior. Analyses were run for 3,000,000 generations, sampling trees and model parameters every 300 generations. Convergence of results was assessed by visual inspection of the log file using Tracer software [59] and accordingly a burn-in period of 300,000 generations (10%) was established. We used TreeAnnotator software (distributed as part of the BEAST software package) to recover the maximum clade credibility (MCC) consensus tree from the post-burn-in sample of trees. The robustness of the inferred clades was evaluated based on Bayesian posterior probabilities (BPPs). Candidate sequences were identified as orthologs when they grouped in a clade with high statistical support (BPP > 0.95) with sequences of known identity.

### Cloning, sequencing and RNA probe generation

Embryos from *L. gigantea* were handled and stored in the freezer as described in Grande and Patel [60]. Embryos from *C. fornicata* were collected and reared as previously described (see, for instance, Henry et al. [49]). High-quality total RNA from embryos of the gastropods *L. gigantea* and *C. fornicata* was extracted using TriZol and purification methods followed the manufacturer’s suggested protocol. The purity and concentration of total RNA was verified with a NanoDrop ND-1000 Spectrophotometer (Thermo Scientific, Wilmington, DE) and approximately 1 µg of total RNA from each developmental stage was used to synthesize cDNA (iScript cDNA Synthesis kit, Bio-Rad, Hercules, CA). Gene-specific primers were designed for each gene (Additional file 4: Table S2). PCR amplification reactions were performed with Phusion HF DNA polymerase, Q5 high-fidelity DNA polymerase and Q5 high GC enhancer buffer (New England Biolabs, Ipswich, MA), according to the manufacturer’s suggested ratios. Amplified PCR products were run on 1% agarose gels, gel-purified (GeneClean Turbo kit, MP Biomedicals, Solon, OH) and cloned into pGem-T Easy vector (Promega, Madison, WI). Digoxigenin (DIG)-labeled and fluorescein (FITC)-labeled antisense and sense RNAs were synthesized from purified pGEM-T easy plasmid DNA that was amplified using SP6 and T7 primers via PCR. Template DNA was used with SP6 and T7 RNA polymerase (Invitrogen, Carlsbad, CA, USA), DIG and fluorescein labeling mix (Roche, Indianapolis, IN, USA) to generate probes. Reactions were purified with RNeasy MinElute Cleanup kit (Qiagen, Valencia, CA), and probe concentrations were verified using a NanoDrop ND-1000 Spectrophotometer.

### In situ hybridization and Hoechst staining

The in situ hybridization protocol for single-gene labeling was modified from Finnerty et al. [61] and Perry et al. [46]. The double labeling in situ protocol in *L. gigantea* was performed as described in Grande and Patel [60]. Embryos were counterstained with 1:10,000 dilution of Hoechst in 80% glycerol/20% 1× PBS for 1 h to visualize nuclei, followed by three washes in PBS and stored in 80% glycerol/20% 1× PBS.

### Microscopy and image analysis

Fixed embryos processed for in situ hybridization were mounted on Rain-X coated (ITW Global Brands, Houston, TX) glass slides in 80% glycerol/20% 1X PBS. Coverslips were prepared as described in Lyons et al. [62] for *C. fornicata* and with two additional supporting coverslips at the sides for *L. gigantea.* All images were acquired using an Axioskop 2 plus microscope (Zeiss) in conjunction with a CoolSnap FX color camera (Roper Scientific) and MetaVue 5.07 (Universal Imaging) software from Centro de Biología Molecular Severo Ochoa SMOC facilities. Additional image processing was done with Helicon Focus (Helicon Soft Ltd. Kharkov, Ukraine) to combine multifocal stacks of images and get focused images and Adobe Photoshop (Adobe Systems Software Ireland Ltd.) for the stacking of bright-field and fluorescent images of *C. fornicata* embryos.

## Results

### Phylogenetic analyses

One single potential Sal1 ortholog was found in several Spiralia, including mollusks, nemerteans, annelids, brachiopods, bryozoans and platyhelminthes (Additional file 1: Table S1). One single potential ortholog was also found in some ecdysozoan groups, such as priapulids, and arthropods (except Diptera, which has two well-known paralogs: Salm and Salr) (Additional file 1: Table S1). In addition, one single ortholog was identified for some other bilaterians, such as acoels, nemertodermatids and *Xenoturbella* (Additional file 1: Table S1). Deuterostomes except vertebrates also have one Sall ortholog (Additional file 1: Table 1). Searches in the databases from non-bilaterian metazoans did not retrieve any potential orthologs (Additional file 1: Table S1).

In order to assign orthology, we first performed a phylogenetic analysis including the newly determined Sall sequences along with previously reported Sall sequences and the related zinc-finger proteins Schnurri, PRDII-BF1 and HIVEP1 (Additional file 5: Fig. S3). The resulting tree confirmed with high statistical support that the newly identified Sall sequences were indeed more closely related to the previously reported Sall sequences than to other zinc-finger-related sequences (BPP = 1) (Additional file 5: Fig. S3). Once orthology of the Sall proteins was established, we performed a second phylogenetic analysis including all Sall orthologs (Additional file 6: Fig. S4). This analysis pointed out the existence of three highly divergent sequences: the platyhelminth Sall, the nematode SEM-4 and the bryozoan Sall proteins (Additional file 6: Fig. S4). All vertebrate Sall sequences formed a clade (BPP = 1) with one supported group for each paralog (Sall1, Sall2, Sall3 and Sall4) (Additional file 6: Fig. S4). All ecdysozoan Sall sequences but SEM4 formed a single clade (BPP = 0.91) (Additional file 6: Fig. S4). Finally, all spiralian Sall sequences, except the platyhelminth, the bryozoan and the annelid Sall, formed a clade (BPP = 1) (Additional file 6: Fig. S4).

### Analysis of Sall protein domains

**Fig. 2:**
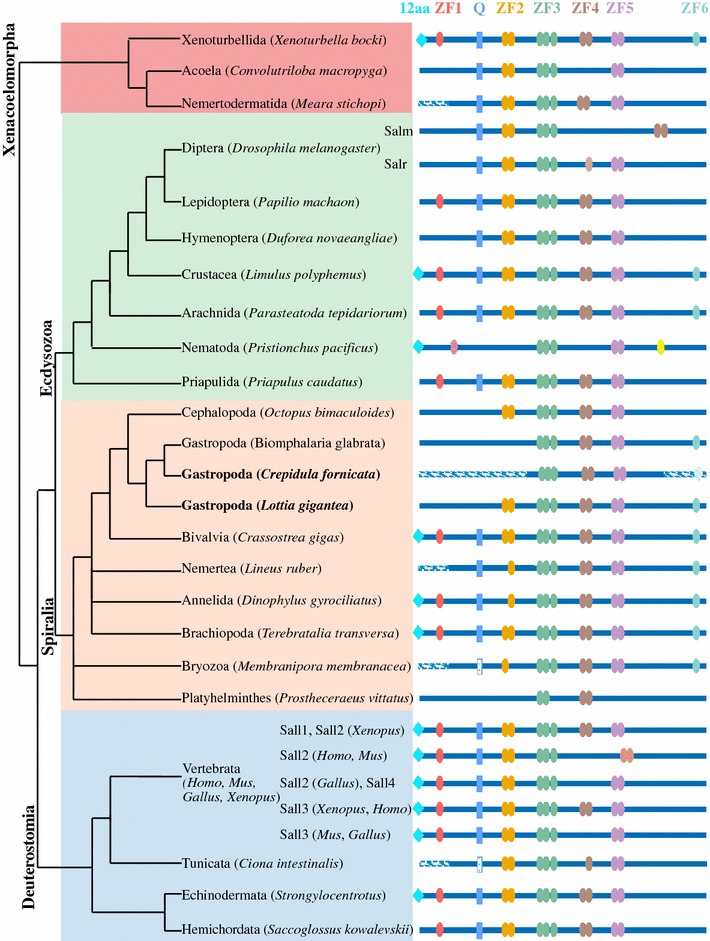
Organization of conserved domains in Sall proteins in Bilateria. The light blue diamond represents the 12 conserved amino acids at the N-terminal end that interact with the HDC NuRD [14]. Colored ovals represent the zinc-finger motifs from ZF1 to ZF6. The blue rectangle represents the poly-Q region. The striped lines represent missing sequence. Phylogenetic tree based on Cannon et al. [63] and Peters et al. [64]. The blue box on the tree highlights the deuterostomes; the orange box, the spiralians; the green box, the ecdysozoans; and the red box, the xenacoelomorphs. Bold names highlight the snail species studied in more detail in this work

All Sall orthologs were aligned in order to identify the protein domains. Gene sequences were incomplete in the gastropod *C. fornicata*, the nemertodermatid, the nemertean, the bryozoan and the tunicate (Fig. 2). The analysis of the protein domains showed that the molecular structure of Sall proteins is highly consistent in bilaterians with some exceptions discussed below (Fig. 2). The ZF domain located in the N-terminal part of the protein (ZF1) corresponds to the C2HC class, and we found it in ecdysozoans, spiralians and deuterostomes, as well as in *Xenoturbella*, a member of the clade Xenacoelomorpha, the sister group of all bilaterians [63] (Fig. 2). The 12 amino acid sequence responsible for the interaction with the histone deacetylase complex NuRD is similarly conserved, but lost in some groups (Fig. 2; Additional file 7: Fig. S5). The poly-Q region is highly conserved, only lost in the nematode SEM-4, the platyhelminth and most mollusks (gastropods and the cephalopod), and partially conserved in the bryozoan and the tunicate (Fig. 2; Additional file 7: Fig. S5). The other ZF domains (2–5) correspond to the C2H2 class arranged in pairs connected by an H/C link and are highly conserved through the evolution of Bilateria (Fig. 2). The second zinc finger (ZF2) is more conserved and contains the characteristic Sal-box. The ZF2 domain is lost in the nematode, the platyhelminth and the gastropod *B. glabrata,* and shows modifications in the nemertean and the annelid, which have lost the N-terminal zinc finger of the pair and the bryozoan, which has lost the C-terminal zinc finger (Fig. 2; Additional file 7: Fig. S5). The ZF3 domain is the most conserved in all groups analyzed here, and it has an additional zinc-finger-associated domain, except in Platyhelminthes, which may have lost it (Fig. 2; Additional file 7: Fig. S5). The ZF4 domain is lost in acoels and in the nematode. Sall2 of all vertebrates, as well as Sall3 of chick and murine, also lack ZF4, while it is modified in tunicates (*Ciona intestinalis*), which have lost the N-terminal zinc finger (Fig. 2; Additional file 7: Fig. S5). The ZF5 is also well conserved, only lost in *Drosophila* Salm, the platyhelminth, and human and murine Sall2 (Fig. 2; Additional file 7: Fig. S5). Our analyses show the presence of a new ZF domain, ZF6, in the C-terminal region. This previously undescribed ZF is present in Xenoturbellida, some ecdysozoans (Crustacea, Arachnida) and spiralians (Mollusca, Nemertea, Annelida, Brachiopoda and Bryozoa) although it is missing in the deuterostomes (Fig. 2; Additional file 7: Fig. S5). This ZF6 domain is confirmed by a single zinc finger that corresponds to the C2H2 class and it does not present a Sal-box.

There are some extra non-homologous ZF domains. Nematodes present a ZF domain with a single zinc finger in the N-terminal region that is not homologous to ZF1 and another single ZF in the C-terminal region. *Drosophila* Salr contains an extra ZF (ZFX) between ZF3 and ZF5 with a single zinc finger, not homologous to ZF4. Human and murine Sall2 present a ZF in the C-terminal region formed by a pair of zinc fingers that are not homologous to ZF4 or ZF5 (Fig. 2).

### Expression of *sall* in *Crepidula fornicata*

**Fig. 3:**
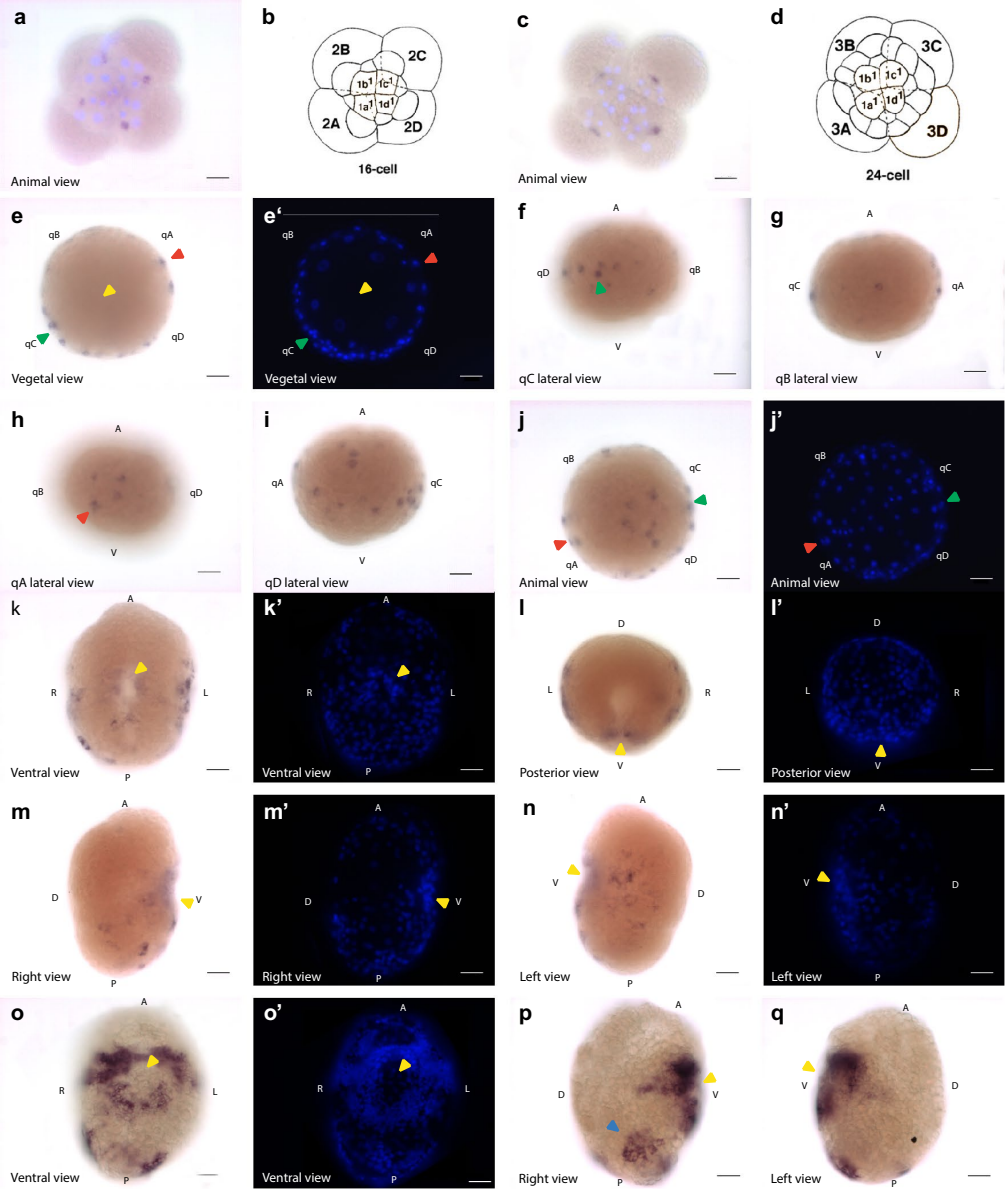
*Sall* expression in *C. fornicata*. *In situ* hybridization of s*all* mRNA in embryos from 16-cell stage to organogenesis. The distribution of labeled mRNA is shown by the dark blue staining. In all images orientation is indicated at the bottom left corner of each panel. Scale bar at the bottom right corner equals 40 µm. Yellow arrowhead indicates the blastopore/stomodeum. **a**, **c** 16- and 24-cell stage embryos, respectively. mRNA is expressed in the macromeres. The lighter colored nuclei are labeled with Hoechst (blue). **b**, **d** Schematic representation of 16- and 24-cell embryos, respectively, in animal view (modified from Henry et al., [47]). Capital letters indicate the macromeres and lowercase letters the micromeres. **e**–**j**′ Localization of *sall* mRNA in blastula stage. **e**–**e**′, **j**–**j**′ Bright-field and fluorescent Hoechst-labeled corresponding images. **f**–**i** Lateral view of each quadrant. Green and red arrowheads indicate stained reference cells in quadrants C and A, respectively, in different views. *q* quadrant, *A* animal pole, *V* vegetal pole. **k**–**n**′ Localization of *sall* mRNA in gastrula stage. Each bright-field image has a corresponding fluorescent Hoechst-labeled image on the right. **o**–**q**′ Localization of *sall* mRNA in organogenesis. **o**, **o**′ Bright-field and fluorescent Hoechst-labeled corresponding images. **p** Bright-field right view. The blue arrow indicates the asymmetric patch of expression present in this side. **q** Bright-field left view. A: anterior; P: posterior; V: ventral; D: dorsal; R: right; L: left

We examined the spatiotemporal expression patterns of the *C. fornicata sall* gene using single whole-mount in situ transcript hybridization and PCR. *C. fornicata* s*all* mRNA does not exhibit maternal expression (Additional file 8: Fig. S6), being detected as soon as the 16-cell to 24-cell stages in all four macromeres of the embryo (Fig. 3a, c). The expression is turned off at the same time in the macromeres during the early blastula stage and transcription starts in a discrete fashion in some cells at the animal pole and in a lateral stripe (Fig. 3e–j), with more cells stained at the C quadrant (Fig. 3f), less at the A and D quadrants (Fig. 3h, i), and with only one single cell stained at the B quadrant (Fig. 3g).

At the gastrula stage, *sall* is expressed in a lateral stripe at the middle–posterior region of the right side and the middle region of the left side and at both sides of the stomodeum (Fig. 3k–n). A similar pattern is observed later during organogenesis, at both sides of the stomodeum, with extended expression on the lateral sides, forming a stripe over the stomodeum (Fig. 3o–q). There is also expression on the posterior region of both the dorsal and right sides (Fig. 3p), where it presents as an asymmetric patch, and this expression is absent in the left side (Fig. 3q).

### Expression of *sall* in *Lottia gigantea*

**Fig. 4:**
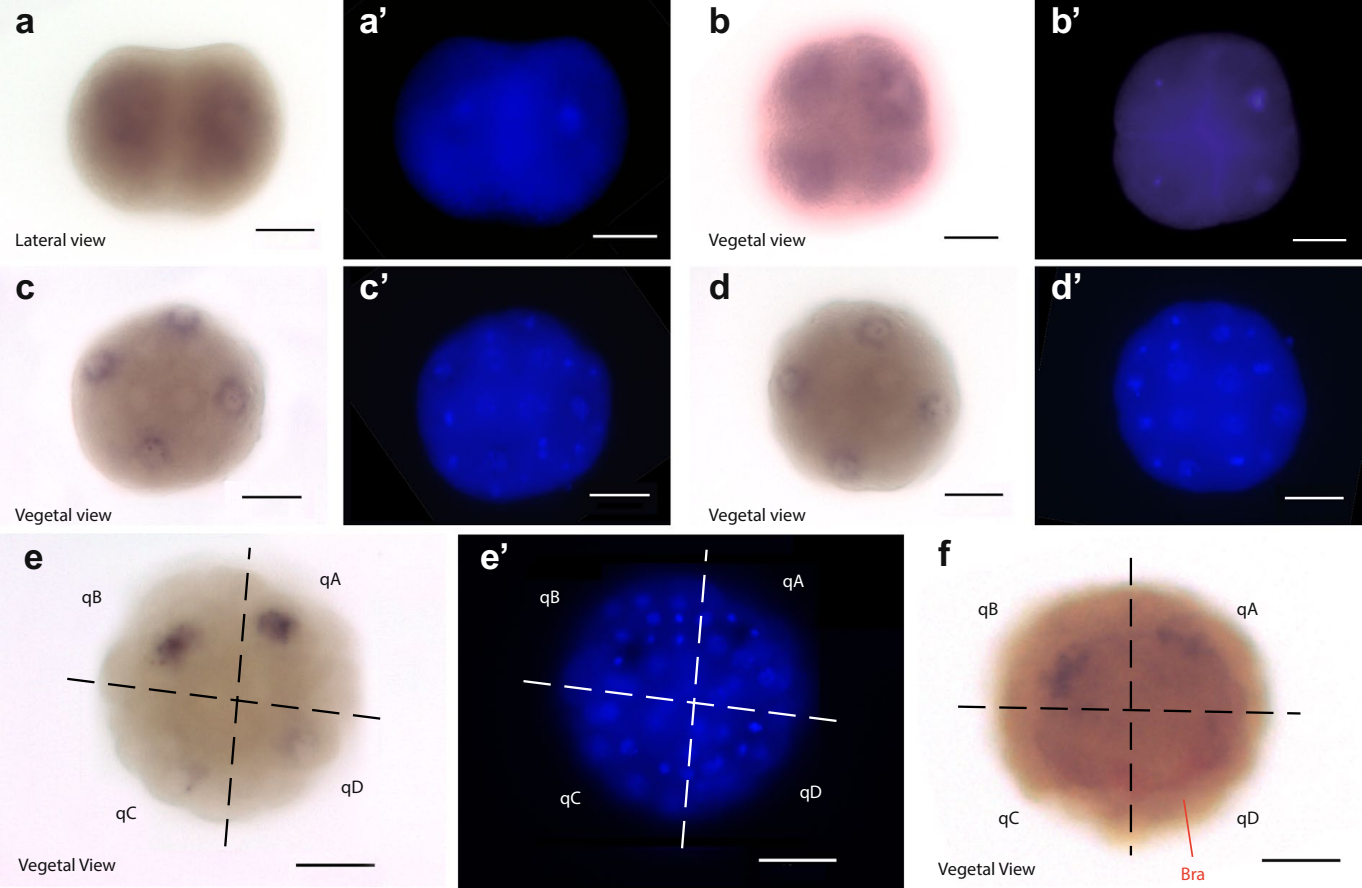
Expression of *sall* during early cleavage in *L. gigantea*. *In situ* hybridization of *sall* mRNA in embryos from 2-cell stage to blastula stage. Each bright-field image has its corresponding fluorescent Hoechst-labeled image at the right. The distribution of labeled mRNA is shown by the dark blue staining. Orientation is indicated at the bottom left corner of each panel. Scale bar at the bottom right corner equals 40 µm. **a**–**a**′ Expression in all cells at 2-cell stage. **b**–**b**′ Expression in all cells at 4-cell stage. **c**–**c** Expression in the 2 m micromeres at 16-cell stage. **d**–**d**′ Expression in the 2 m micromeres at 24-cell stage. **e**–**e**′ Expression of *sall* mRNA at blastula stage. **f** Double in situ hybridization of *sall* (dark blue) and *brachyury* (red). Indicated in red, labeled *brachyury* mRNA in the D quadrant. Note that *sall* expression is restricted to the A and B quadrants. q: quadrant; bra: *brachyury*. Quadrants are separated by dashed lines in **e**, **f**

**Fig. 5:**
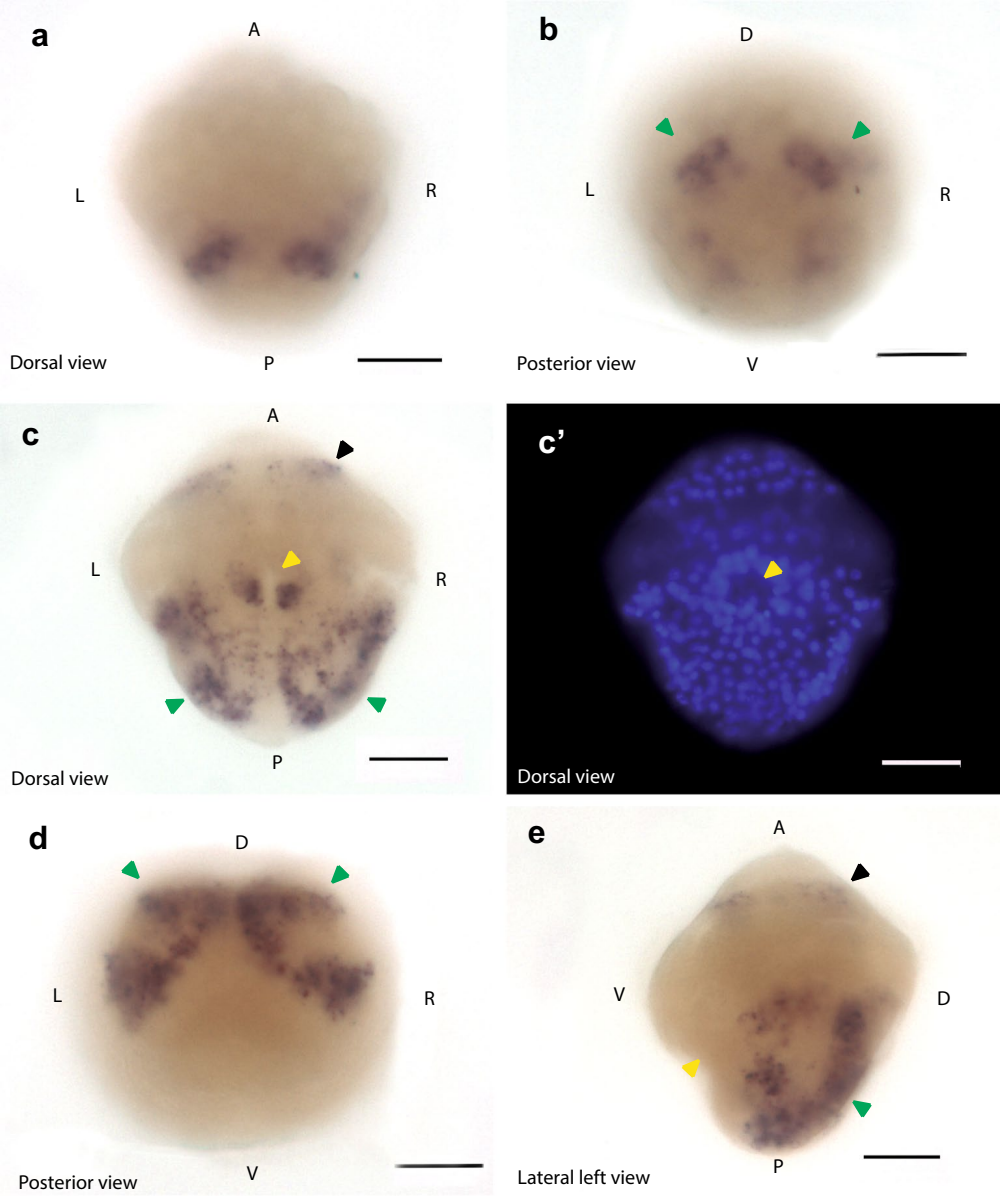
Expression of *sall* during trochophore stages in *L. gigantea*. The distribution of the labeled mRNA is shown by the dark blue staining. Orientation is indicated at the bottom left corner of each panel. Scale bar at the bottom right corner equals 40 µm. A: anterior; P: posterior; V: ventral; D: dorsal; R: right; L: left. **a**, **b** Localization of *sall* mRNA in pre-trochophore stage. Expression starts to extend from the progeny of 2 m micromeres. **c**–**e** *In situ* hybridization of *sall* mRNA at trochophore stage. Black arrowhead indicates the stained ring in the cephalic region. Green arrowhead indicates the dorsolateral ectodermic strip. Yellow arrowhead indicates the stomodeum

We also examined the spatiotemporal expression pattern of the *L. gigantea sall* gene using single and double whole-mount in situ transcript hybridization. *L. gigantea* shows maternal expression of *sall*, detected in all cells in eggs, 2-cell and 4-cell embryos (Fig. 4a, b). However, *sall* mRNA is differentially segregated since expression at the 8-cell stage is restricted to macromeres, and at 16-cell and 24-cell stages, this mRNA is detected exclusively in the 2 m micromeres of each quadrant (Additional file 9: Fig. S7; Fig. 4c, d). *L. gigantea* presents equal cleavage, and prior to the 32-cell stage, the embryo is radially symmetrical and so is the expression pattern of *sall*. After the 32-cell stage when zygotic expression starts, *sall* expression remains in two quadrants, while the expression decays in the other two, resulting in an asymmetric pattern (Fig. 4e, f). As the embryo is radially symmetrical and in order to clarify in which quadrants the expression is reduced, a double whole-mount in situ hybridization was performed with *sall* and *brachyury*, a gene known to be involved in the establishment of the anteroposterior axis and which expression is restricted to the D quadrant [60, 65] (Fig. 4f). The results show that the expression of *Lottia sall* is maintained in A and B quadrants and decays in C and D quadrants after the 32-cell stage during blastula and gastrula stages, but is still restricted to the same cells and their progeny (Fig. 4e, f). The progeny of those cells that expressed *sall* in the A and B quadrants at the 32-cell stage maintained the expression of *sall* up to the larva stage (Fig. 5). Finally, in the trochophore stage *sall* is also expressed in a ring in the cephalic region (Fig. 5c, e) and in a dorsolateral ectodermic strip in the post-trochal region at both sides of the trochophore (Fig. 5c–e).

## Discussion

Our study is the first to investigate Sall proteins in a broad phylogenetic framework. Previous studies had identified Sall proteins exclusively in nematodes, flies, planarians and vertebrates. Here we showed that *sall* is also present in members of all main groups across Bilateria (i.e., Xenacoelomorpha, Ecdysozoa, Spiralia and Deuterostomia) (Fig. 2). In addition, we could not find any Sall protein in the genome of non-bilaterians (the cnidarian *Nematostella vectensis*, the ctenophore *Pleurobrachia bachei*, the placozoan *Trichoplax adhaerens* and the sponge *Amphimedon queenslandica*), which suggests that Sall proteins might be an exclusive feature of bilaterians.

The analysis of the Sall protein domains and their comparison among bilaterians showed that orthologous zinc fingers in different Sall proteins are more similar in sequence among them than to other zinc-finger domains of the same Sall protein. This may indicate that the characteristic arrangement of the multiple double zinc fingers is not the result of independent duplications from a unique double zinc finger in different organisms, but is instead the ancient arrangement of this family of transcription factors. The novel zinc-finger domain described here (ZF6) is present in Ecdysozoa, Spiralia and Xenacoelomorpha, and one can interpret this as being plesiomorphic for all bilaterian Sall proteins and then secondarily lost in some specific groups like deuterostomes, nematodes, priapulids and insects (Fig. 2; Additional file 7: Fig. S5). Similarly, we found the ZF1 domain in most phyla, implying that it may also be an ancestral domain in bilaterian Sall proteins, and not a domain exclusive of the vertebrate homologs, as previously thought [12] (Fig. 2). In summary, this new evidence suggests that the bilaterian common ancestor probably had a Sall protein with at least 6 zinc-finger domains.

From this ancestral Sall protein, the evolution of Sall proteins in bilaterians might have occurred mostly as a result of the loss of protein domains and gene duplications leading to diversification of functions in paralogs, as it occurs in vertebrate or *Drosophila* Sall proteins. For instance, the presence/absence/modification of either ZF4 or ZF5 in paralogs of chordates and *Drosophila* might be related to the differential repression or activation mechanisms of these Sall proteins [10]. The ZF4 and ZF5 domains are necessary for the localization to heterochromatin in vertebrates [64]. In addition, this family of zinc fingers is capable of binding to DNA at specific AT-rich regions [16], as do other known zinc-finger proteins [16, 65], which is further emphasized by the occurrence of alternative splicing in this region [9, 66]. The variability in this region suggests that it might mediate binding specificity. Repression and activation capacities could reside in different Sall proteins in chordates and *Drosophila* since they have several paralogs [12] with different configurations in this region (Fig. 2). Both capacities could exist within the same protein in other groups that have a single ortholog with both ZF4 and ZF5 (Fig. 2). Therefore, Sall proteins might bind to specific sequences and cell-specific protein partners, resulting in different Sall protein conformations and thus exposing either repression or activation domains [11]. The most extreme cases of the loss of protein domains are the platyhelminth Sall, which only retains ZF3 and ZF4, and the nematode SEM-4, which retains ZF3 and ZF5, but also possesses two unique ZF domains (Fig. 2).

The 12 N-terminal amino acid sequence of the Sall protein is responsible for the repression activity through the recruitment of the histone deacetylase complex NuRD [14], but its function is correlated with its localization in the nucleus, which might be dependent on the ZF1 [13]. The almost absolute correlation between the presence and absence of both motifs observed in the analysis of the protein domains (Fig. 2; Additional file 7: Fig. S5) could be explained by their functions, as the repression through NuRD would not be possible without the localization provided by the ZF1. Another region located between ZF2 and ZF3 is a putative repression and heterochromatin localization domain [15] in vertebrate Sall and its functions might be conserved during evolution. These ZFs are encoded in a single large exon [1], show high identity and are especially well conserved, with ZF3 being conserved in all species analyzed (Fig. 2; Additional file 7: Fig. S5), and they can be easily homologized. However, ZF2 is not present in SEM-4 and *Biomphalaria* Sall (Fig. 2; Additional file 7: Fig. S5). Also, in contrast, in *Drosophila* Salm and Salr there is an activation domain located between the poly-Q and ZF3 domains [12]. In addition, the repression activity does not appear to be related to ZF3, which is able to bind to DNA. Rather, repression may be related to the region located at the N-terminal side of ZF3 [15], where we observed a highly conserved sequence in all species analyzed (consensus sequence: SETSKLQQLVENID). One more region, the poly-Q domain, could be important for the biological activity of these proteins, making possible the interaction among the paralogs as suggested by Sweetman et al. (2003) [23]. However, our data show that the poly-Q domain is not only restricted to vertebrates, but is widely present across the Bilateria, even in species with only one Sall form. Therefore, further studies are needed to understand the role of the poly-Q domain in Sall proteins.

The best homology among the double zinc fingers was found in the eight amino acids of the “Sal-box,” which are present in all C-terminal finger motifs of the ZF pairs and the H/C link [3]. Interestingly, in all cases where one finger of a pair is lost, it is the N-terminal. This kind of zinc-finger pair has also been observed in other proteins: the human transcription factor PRDII-BF1 [5] and *Drosophila* Schnurri [4, 5]. Sequence similarity within the pairs of zinc fingers suggests the importance of the Sal-box for the structure of the motif and may define a conserved subfamily of zinc-finger proteins.

Given the implication of *sall* in the development of *Drosophila* [11], *C. elegans* [8], *S. mediterranea* [9] and vertebrates [12], we studied the expression pattern through the development of two snail species: *C. fornicata* (Fig. 3) and *L. gigantea* (Figs. 4, 5) for a better understanding of the relevance of this protein and its functions in animal development. In both species, *sall* was expressed through most of the development process, suggesting that *sall* has a role in the development of spiralians, as in other bilaterians.

However, the differences in the expression patterns found in the snails *L. gigantea* and *C. fornicata* indicate that the degree of conservation of *sall* coding sequences does not necessarily imply similarities of expression patterns (Figs. 3, 4, 5). Interestingly, *L. gigantea* show maternal expression of *sall*, a feature that was previously described only in murine *sall4,* which presents maternal mRNA at the 2-cell stage, but is degraded in the next cleavage [67]. While *Lottia* shows a high level of maternal expression in all cells at the 2-cell and 4-cell stages and in the 2 m micromeres at early cleavage, this does not seem to be the case for *Crepidula* (Figs. 3, 4). The appearance of *sall* mRNA at the 16-cell stage in *Crepidula* embryos implies that *sall* mRNA is not maternal in this species and that zygotic expression of *sall* begins later, at the 16-cell stage (Fig. 3). Further work in other bilaterian embryos will help elucidate whether maternal expression is an ancestral feature of Sall proteins.

The transcriptional expression of *sall* differs greatly between *Lottia* and *Crepidula* during gastrulation and early organogenesis stages, although there are some common patterns observed in larvae (Figs. 3, 4, 5). The restriction in the expression pattern to A and B quadrants seen in *Lottia* was not previously described for any other gene in this species (Fig. 4). By contrast, in *Crepidula*, the expression is mainly restricted to A, C and D quadrants (Fig. 3). The lateral asymmetries in the expression pattern in the posterior region in *C. fornicata* during organogenesis (Fig. 3) might be related to the differential proliferation during shell/mantle growth as previously described for other genes [46]. However, in both species *sall* is expressed close to the stomodeum in the posterior lateral ectoderm (Figs. 3, 4, 5). In addition, in *Lottia sall* is expressed in a ring in the cephalic domain (Fig. 5).

Determination of functional conservation of Sall proteins across bilaterians would require further experiments in additional groups. However, several lines of published evidence indicate that at least some functions of *sall* have been evolutionarily conserved. For instance, the *sall* genes appear to function as cell fate determinants, regulators of Hox genes and AP patterning, and as transcriptional repressors. In addition, their function in neural development seems to be conserved. In *Drosophila*, Sall is required in neuronal precursors and differentiated neurons to restrict neuronal fates to the proper cells [11], SEM-4 controls neural development in *C. elegans* [8] and the *sall* genes are involved in determining neural fates in chordates [20, 35]. Here we have shown that in *L. gigantea sall* is expressed in the cephalic region (Fig. 5), which may reflect its role in neural fate specification, although additional experiments are needed to confirm this hypothesis. If new evidence corroborates this neural expression in Spiralia, this may support the potential ancestral role of Sall proteins in neuronal development. An interesting question would be whether *Sall* is expressed in *C. fornicata* nephridia during organogenesis, since Sall1 is necessary in mice to develop kidneys [37, 38] and the human SALL1 is mutated in patients with Townes–Brocks syndrome (TBS) [68], which produces among other symptoms abnormal kidney development. Even in planarians, Sall is required for protonephridia regeneration [9].

## Conclusions

The results of our analyses provide novel evidence about the evolution of Sall proteins and their functional domains. Specifically, they show that *sall* is conserved across Bilateria and might be exclusive to this group. The ancestral Sall protein probably presented six zinc-finger domains, including a sixth zinc-finger domain that is reported here for the first time (ZF6, Fig. 2).

We also present the first report of *sall* gene expression in snails; the results highlight its importance in the development of bilaterians. *sall* has maternal expression and is expressed in the ectoderm, surrounding the stomodeum and in a cephalic ring in snails. The expression in the cephalic region in snails suggests a possible ancestral role of *sall* in neural development in bilaterians.

## Additional files

**Additional file 1: Table S1.** Protein sequences used in this study with their length, accession number and the corresponding species.

**Additional file 2: Fig. S1.** Sequence alignment of Sal proteins. Alignment of the deduced amino acid sequences of Sall proteins of different taxa. Amino acids shared by more than 50% of the species are shaded in gray

**Additional file 3: Fig. S2.** Sequence alignment of Sal proteins. Alignment of the deduced amino acid sequences of Sall proteins of different taxa. Amino acids shared by more than 50% of the species are shaded in gray.

**Additional file 4: Table S2.** Primer sequences used in this study.

**Additional file 5: Fig. S3.** Phylogenetic analysis of zinc-finger proteins containing the “Sal-box” (FTTKGNLK). Maximum clade credibility tree from the BEAST analysis including the deduced amino acid sequences of Sall proteins together with Schnurri, PRDII-BF1 and HIVEP1. Numbers indicated above the nodes are Bayesian posterior probabilities (BPP). The yellow box highlights Sall sequences across the Bilateria (BPP = 1) and the red box, the sequences of the other zinc-finger-related proteins (BPP = 1).

**Additional file 6: Fig. S4.** Phylogenetic analysis of Sall proteins across the Bilateria. Maximum clade credibility tree from the BEAST analysis including the deduced amino acid sequences of Sall proteins. Numbers above nodes are Bayesian posterior probabilities. The dark blue line groups Sall sequences of Vertebrates; the light blue line, the sequences of deuterostomes; the green line, the sequences of ecdysozoans (E); the orange line, the sequences of spiralians (S); and the red line, the sequences of xenacoelomorphs (X).

**Additional file 7: Fig. S5.** Hypothesized Sall protein gains and losses during Bilateria evolution. The turquoise diamond represents the 12 conserved amino acids at the N-terminal end that interact with the HDC NuRD [14]. Colored ovals represent the zinc-finger motifs from ZF1 to ZF6. The blue rectangle represents the poly-Q region. The striped lines represent missing sequence. Phylogenetic tree based on Cannon et al. [63] and Peters et al. [64]. The blue box on the tree highlights the deuterostomes; the orange box, the spiralians; the green box, the ecdysozoans; and the red box, the xenacoelomorphs. Bold names highlight the snail species studied in more detail in this work. On each branch, proposed gains and losses of Sall protein domains are indicated.

**Additional file 8: Fig. S6.** Amplification of *spalt* from cDNA extracted from zygotes and 24-h post-fertilization *Crepidula* embryos. Amplification of *spalt* (*Cfo*-*spalt*) was only detected using cDNA from 24-h post-fertilization *Crepidula* embryos. There was no amplification at all when cDNA from zygotes was used. The gene *twist* from *Crepidula* (*Cfo*-*twist*) was used as a positive control since *twist* is detected at both stages of development. PCR products were run on 1% agarose gels.

**Additional file 9: Fig. S7.** Schematic drawings of *sall* expression at 4-cell, 8-cell, 16-cell stages and trochophore larva in *L. gigantea*. a: expression in all cells at 4-cell stage. b: Expression in the macromeres at 8-cell stage. c: Expression in the 2 m micromeres at 16-cell stage. Micromere 2a underlined in green and 2b underlined in orange. d: expression in the trochophore. In green and orange, expression of *sall* in the dorsolateral ectoderm derived from 2a and 2b, respectively. In pink *sall*, expression in the cephalic ring and next to the stomodeum, not derived from 2a and 2b micromeres. Drawings modified from Dictus and Damen [69].

## References

[1] Kuhnlein RP, Frommer G, Friedrich M, Gonzalez-Gaitan M, Weber A, Wagner-Bernholz JF, Gehring WJ, Jackle H, Schuh R (1994). Spalt encodes an evolutionarily conserved zinc finger protein of novel structure which provides homeotic gene function in the head and tail region of the *Drosophila* embryo. EMBO J.

[2] Frei E, Schuh R, Baumgartner S, Burri M, Noll M, Jurgens G, Seifert E, Nauber U, Jackle H (1988). Molecular characterization of spalt, a homeotic gene required for head and tail development in the *Drosophila* embryo. EMBO J.

[3] Schuh R, Aicher W, Gaul U, Cote S, Preiss A, Maier D, Seifert E, Nauber U, Schroder C, Kemler R (1986). A conserved family of nuclear proteins containing structural elements of the finger protein encoded by Kruppel, a *Drosophila* segmentation gene. Cell.

[4] Arora K, Dai H, Kazuko SG, Jamal J, O’Connor MB, Letsou A, Warrior R (1995). The *Drosophila* schnurri gene acts in the Dpp/TGF signaling pathway and encodes a transcription factor homologous to the human MBP family. Cell.

[5] Grieder NC, Nellen D, Burke R, Basler K, Affolter M (1995). Schnurri is required for *Drosophila* Dpp signaling and encodes a zinc finger protein similar to the mammalian transcription factor PRDII-BF1. Cell.

[6] Fan CM, Maniatis T (1990). A DNA-binding protein containing two widely separated zinc finger motifs that recognize the same DNA sequence. Genes Dev.

[7] Barrio R, Shea MJ, Carulli J, Lipkow K, Gaul U, Frommer G, Schuh R, Jäckle H, Kafatos FC (1996). The spalt-related gene of *Drosophila melanogaster* is a member of an ancient gene family, defined by the adjacent, region-specific homeotic gene spalt. Dev Genes Evol.

[8] Basson M, Horvitz HR (1996). The *Caenorhabditis elegans* gene *sem*-*4* controls neuronal and mesodermal cell development and encodes a zinc finger protein. Genes Dev.

[9] Scimone ML, Srivastava M, Bell GW, Reddien PW (2011). A regulatory program for excretory system regeneration in planarians. Development.

[10] Sweetman D, Münsterberg A (2006). The vertebrate *spalt* genes in development and disease. Dev Biol.

[11] de Celis JF, Barrio R (2009). Regulation and function of Spalt proteins during animal development. Int J Dev Biol.

[12] Sánchez J, Talamillo A, González M, Sánchez-Pulido L, Jiménez S, Pirone L, Sutherland JD, Barrio R (2011). *Drosophila* Sal and Salr are transcriptional repressors. Biochem J.

[13] Kiefer SM, McDill BW, Yang J, Rauchman M (2002). Murine Sall1 represses transcription by recruiting a histone deacetylase complex. J Biol Chem.

[14] Lauberth SM, Rauchman M (2006). A conserved 12-amino acid motif in Sall1 recruits the nucleosome remodeling and deacetylase corepressor complex. J Biol Chem.

[15] Netzer C, Bohlander SK, Hinzke M, Chen Y, Kohlhase J (2006). Defining the heterochromatin localization and repression domains of SALL1. Biochim Biophys Acta (BBA) Mol Basis Dis.

[16] Yamashita K, Sato A, Asashima M, Wang PC, Nishinakamura R (2007). Mouse homolog of SALL1, a causative gene for Townes–Brocks syndrome, binds to A/T-rich sequences in pericentric heterochromatin via its C-terminal zinc finger domains. Genes Cells.

[17] Netzer C, Rieger L, Brero A, Zhang CD, Hinzke M, Kohlhase J, Bohlander SK (2001). SALL1, the gene mutated in Townes–Brocks syndrome, encodes a transcriptional repressor which interacts with TRF1/PIN2 and localizes to pericentromeric heterochromatin. Hum Mol Genet.

[18] Li D, Tian Y, Ma Y, Benjamin T (2004). p150Sal2 is a p53-independent regulator of p21WAF1/CIP. Mol Cell Biol.

[19] Wu Q, Chen X, Zhang J, Loh YH, Low TY, Zhang W, Sze SK, Lim B, Ng HH (2006). Sall4 interacts with Nanog and co-occupies Nanog genomic sites in embryonic stem cells. J Biol Chem.

[20] Young JJ, Kjolby RA, Kong NR, Monica SD, Harland RM (2014). Spalt-like 4 promotes posterior neural fates via repression of pou5f3 family members in Xenopus. Development.

[21] Zhang J, Tam WL, Tong GQ, Wu Q, Chan HY, Soh BS, Lou Y, Yang J, Ma Y, Chai L, Ng HH, Lufkin T, Robson P, Lim B (2006). Sall4 modulates embryonic stem cell pluripotency and early embryonic development by the transcriptional regulation of Pou5f1. Nat Cell Biol.

[22] Lauberth SM, Bilyeu AC, Firulli BA, Kroll KL, Rauchman M (2007). A phosphomimetic mutation in the Sall1 repression motif disrupts recruitment of the nucleosome remodeling and deacetylase complex and repression of Gbx2. J Biol Chem.

[23] Sweetman D, Smith T, Farrell ER, Chantry A, Münsterberg A (2003). The conserved glutamine-rich region of chick csal1 and csal3 mediates protein interactions with other Spalt family members: implications for Townes–Brocks syndrome. J Biol Chem.

[24] Jürgens G (1988). Head and tail development of the Drosophila embryo involves *spalt*, a novel homeotic gene. EMBO J.

[25] de Celis JF, Barrio R, Kafatos FC (1996). A gene complex acting downstream of *dpp* in *Drosophila* wing morphogenesis. Nature.

[26] Kuhnlein RP, Schuh R (1996). Dual function of the region-specific homeotic gene *spalt* during *Drosophila* tracheal system development. Development.

[27] de Celis JF, Barrio R (2000). Function of the *spalt/spalt*-*related* gene complex in positioning the veins in the Drosophila wing. Mech Dev.

[28] de Celis JF, Barrio R, Kafatos FC (1999). Regulation of the *spalt/spalt*-*related* gene complex and its function during sensory organ development in the *Drosophila* thorax. Development.

[29] Elstob PR, Brodu V, Gould AP (2001). spalt-dependent switching between two cell fates that are induced by the *Drosophila* EGF receptor. Development.

[30] Cantera R, Lüer K, Rusten TE, Barrio R, Kafatos FC, Technau GM (2002). Mutations in *spalt* cause a severe but reversible neurodegenerative phenotype in the embryonic central nervous system of *Drosophila melanogaster*. Development.

[31] Grant K, Hanna-Rose W, Han M (2000). *sem*-*4* promotes vulval cell-fate determination in *Caenorhabditis elegans* through regulation of *lin*-*39* Hox. Dev Biol.

[32] Toker AS, Teng Y, Ferreira HB, Emmons SW, Chalfie M (2003). The *Caenorhabditis elegans* spalt-like gene *sem*-*4* restricts touch cell fate by repressing the selector Hox gene *egl*-*5* and the effector gene *mec*-*3*. Development.

[33] Ott T, Parrish M, Bond K, Schwaeger-Nickolenko A, Monaghan AP (2001). A new member of the spalt like zinc finger protein family, Msal-3, is expressed in the CNS and sites of epithelial/mesenchymal interaction. Mech Dev.

[34] Camp E, Hope R, Kortschak RD, Cox TC, Lardelli M (2003). Expression of three *spalt* (*sal*) gene homologues in zebrafish embryos. Dev Genes Evol.

[35] Onai T, Sasai N, Matsui M, Sasai Y (2004). Xenopus XsalF: anterior neuroectodermal specification by attenuating cellular responsiveness to *Wnt* signaling. Dev Cell.

[36] Buck A, Kispert A, Kohlhase J (2001). Embryonic expression of the murine homologue of SALL1, the gene mutated in Townes–Brocks syndrome. Mech Dev.

[37] Nishinakamura R, Matsumoto Y, Nakao K, Nakamura K, Sato A, Copeland NG, Gilbert DJ, Jenkins NA, Scully S, Lacey DL, Katsuki M, Asashima M, Yokota T (2001). Murine homolog of SALL1 is essential for ureteric bud invasion in kidney development. Development.

[38] Basta JM, Robbins L, Kiefer SM, Dorsett D, Rauchman M (2014). Sall1 balances self-renewal and differentiation of renal progenitor cells. Development.

[39] Farrell ER, Münsterberg AE (2000). *csal1* is controlled by a combination of *FGF* and *Wnt* signals in developing limb buds. Dev Biol.

[40] Kawakami Y, Uchiyama Y, Esteban CR, Inenaga T, Koyano-Nakagawa N, Kawakami H, Belmonte JCI (2009). *Sall* genes regulate region-specific morphogenesis in the mouse limb by modulating Hox activities. Development.

[41] Dunn CW, Hejnol A, Matus DQ, Pang K, Browne WE, Smith SA (2008). Broad phylogenomic sampling improves resolution of the animal tree of life. Nature.

[42] Nordberg H, Cantor M, Dusheyko S, Hua S, Poliakov A, Shabalov I, Smirnova T, Grigoriev IV, Dubchak I. The genome portal of the Department of Energy Joint Genome Institute: 2014 updates. Nucleic Acids Res. 2014; 44(D1), D26–31. http://genome.jgi.doe.gov/Lotgi1/. Accessed 5 May 2016. 10.1093/nar/gkt1069 PMC3965075 24225321

[43] Henry JJ, Perry KJ (2008). MAPK activation and the specification of the D quadrant in the gastropod mollusc, *Crepidula fornicata*. Dev Biol.

[44] Henry JQ, Perry KJ, Martindale MQ (2010). β-catenin and early development in the gastropod, *Crepidula fornicata*. Integr Comp Biol.

[45] Grande C, Martín-Durán JM, Kenny NJ, Truchado-García M, Hejnol A (2015). Evolution, divergence and loss of the Nodal signalling pathway: new data and a synthesis across the Bilateria. Int J Dev Biol.

[46] Perry KJ, Lyons DC, Truchado-Garcia M, Fischer AH, Helfrich LW, Johansson KB, Diamond JC, Grande C, Henry JQ (2015). Deployment of regulatory genes during gastrulation and germ layer specification in a model spiralian mollusc *Crepidula*. Dev Dyn.

[47] Hiebert LS, Maslakova SA (2015). Hox genes pattern the anterior–posterior axis of the juvenile but not the larva in a maximally indirect developing invertebrate, *Micrura alaskensis* (Nemertea). BMC Biol.

[48] Hiebert LS, Maslakova SA (2015). Expression of Hox, Cdx, and Six3/6 genes in the hoplonemertean *Pantinonemertes californiensis* offers insight into the evolution of maximally indirect development in the phylum Nemertea. Evodevo.

[49] Henry JJ, Collin R, Perry KJ (2010). The slipper snail, *Crepidula*: an emerging lophotrochozoan model system. Biol Bull.

[50] Kenny NJ, Truchado-García M, Grande C (2016). Deep, multi-stage transcriptome of the schistosomiasis vector *Biomphalaria glabrata* provides platform for understanding molluscan disease-related pathways. BMC Infect Dis.

[51] Kearse M, Moir R, Wilson A, Stones-Havas S, Cheung M, Sturrock S, Thierer T (2012). Geneious basic: an integrated and extendable desktop software platform for the organization and analysis of sequence data. Bioinformatics.

[52] Altschul SF, Gish W, Miller W, Myers EW, Lipman DJ (1990). Basic local alignment search tool. J Mol Biol.

[53] National Center for Biotechnology Information, U.S. National Library of Medicine 8600 Rockville Pike, Bethesda MD, 20894 USA. https://www.ncbi.nlm.nih.gov/. Accessed 6 May 2016.

[54] Olson SA (1994). MacVector: an integrated sequence analysis program for the Macintosh. Methods Mol Biol.

[55] Larkin MA, Blackshields G, Brown NP, Chenna R, McGettigan PA, McWilliam H, Valentin F, Wallace IM, Wilm A, Lopez R, Thompson JD, Gibson TJ, Higgins DG (2007). Clustal W and Clustal X version 2.0. Bioinformatics.

[56] Drummond AJ, Suchard MA, Xie D, Rambaut A (2012). Bayesian phylogenetics with BEAUti and the BEAST 1.7. Mol Biol Evol.

[57] Jones DT, Taylor WR, Thornton JM (1992). The rapid generation of mutation data matrices from protein sequences. Comput Appl Biosci CABIOS.

[58] Abascal F, Zardoya R, Posada D (2005). ProtTest: selection of best-fit models of protein evolution. Bioinformatics.

[59] Rambaut A, Suchard MA, Xie D, Drummond AJ. Tracer v1.6. (2014). http://beast.bio.ed.ac.uk/Tracer. Accessed 15 June 2016.

[60] Grande C, Patel NH (2009). Nodal signalling is involved in left–right asymmetry in snails. Nature.

[61] Finnerty JR, Paulson D, Burton P, Pang K, Martindale MQ (2003). Early evolution of a homeobox gene: the parahox gene *Gsx* in the Cnidaria and the Bilateria. Evol Dev.

[62] Lyons DC, Perry KJ, Henry JQ (2015). Spiralian gastrulation: germ layer formation, morphogenesis, and fate of the blastopore in the slipper snail *Crepidula fornicata*. EvoDevo.

[63] Cannon JT, Vellutini BC, Smith J, Ronquist F, Jondelius U, Hejnol A (2016). Xenacoelomorpha is the sister group to Nephrozoa. Nature.

[64] Peters RS, Meusemann K, Petersen M, Mayer C, Wilbrandt J, Ziesmann T, Aberer A (2014). The evolutionary history of holometabolous insects inferred from transcriptome-based phylogeny and comprehensive morphological data. BMC Evol Biol.

[65] Lartillot N, Lespinet O, Vervoort M, Adoutte A (2002). Expression pattern of Brachyury in the mollusc *Patella vulgata* suggests a conserved role in the establishment of the AP axis in Bilateria. Development.

[66] Gogos JA, Hsu T, Bolton J, Kafatos FC (1992). Sequence discrimination by alternatively spliced isoforms of a DNA binding zinc finger domain. Science.

[67] Elling U, Klasen C, Eisenberger T, Anlag K, Treier M (2006). Murine inner cell mass-derived lineages depend on Sall4 function. Proc Natl Acad Sci.

[68] Kohlhase J (2000). *SALL1* mutations in Townes-Brocks syndrome and related disorders. Hum Mutat.

[69] Damen P, Dictus WJAG (1994). Cell lineage of the prototroch of *Patella vulgata* (Gastropoda, Mollusca). Dev Biol.

