# Supplementary material for: Molecular, phylogenetic and developmental analyses of Sall proteins in bilaterians
Source: EvoDevo. 2018 Apr 10;9:9. doi: 10.1186/s13227-018-0096-z (PMC5892016; doi:10.1186/s13227-018-0096-z)
Supplement: Supplementary file 2 — Additional file 2: Fig. S1. Sequence alignment of Sal proteins. Alignment of the deduced amino acid sequences of Sall proteins of different taxa. Amino acids shared by more than 50% of the species are shaded in gray [file 13227_2018_96_MOESM2_ESM.pdf]

Position markers: 10, 20, 30, 40, 50, 60, 70, 80, 90, 100, 110

Sequence	Alignment (positions 1–110)
PRDII-BF1 (Xenopus laevis)	KYICEYCSRACAKPSVLLKHIRSHTGERPYPCVTCGFSFKTKSNLYKHKKSHAHAIYICEECGIRCKKPSMLKKHIRTHTDVRPYHCTYCNFSFKTKGNLTKHMKSKAHS
Schnurri (Drosophila melanogaster)	ZINCFINGERPROTEINRYVCQYCNLICAKPSVLEKHIRAHTNERPYPCDTCGIAFKTKSNLYKHCRSRSHAAYVCSECGIRCKKPSMLKKHIRTHTDVRPFTCSHCNFS
HIVEP1 (Homo sapiens)	HUMANIMMUNODEFICIENCYVIRUSTYPEIENHANCERBINDINGPROTEINKYICEYCNRACAKPSVLLKHIRSHTGERPYPCVTCGFSFKTKSNLYKHKKSHAHTIY
SALL1 (Homo sapiens)	PNECIICHRVLSCQSALKMHYRTHTGERPFKCKICGRAFTTKGNLKTHYSVHRAMPTACDICGKTFACQSALDIHYRSHTKERPFICTVCNRGFSTKGNLKQHMLTHQMR
Xsall1 (Xenopus laevis)	PNECVICHRVLSCQSALKMHYRTHTGERPFKCKVCGRAFTTKGNLKTHYSVHRAMPTICDICGKTFACQSALDIHYRSHTKERPFICTVCNRGFSTKGNLKQHMLTHQMR
Sall3 (Homo sapiens)	PNQCVICHRVLSCQSALKMHYRTHTGERPFKCKICGRAFTTKGNLKTHFGVHRAKPTVCGVCGKPFACKSALEIHYRSHTKERPFVCALCRRGCSTMGNLKQHLLTHRLK
Xsal3 (Xenopus laevis)	PNQCVICHRVLSCHSALKMHYRTHTGERPFKCKVCGRAFTTKGNLKTHFGVHRSKPTVCNICGKPFACKSALEIHYRSHTKERPFICTVCKRGCSTMGNLKQHLLTHKLK
Hemichordata (Saccoglossus kowalevskii)	PNQCIICHRILSCKSALQMHYRTHTGERPFKCKLCTRAFTTKGNLKTHYGVHRSKPTMCNICGKAFACASALEIHYRSHTKERPFRCDICVKGFSTKGNLKQHMLTHKIR
Echinodermata (Strongylocentrotus purpuratus)	PNQCVICHRVLSCKSSLQLHYRTHTGERPFRCKICGRSFTTKGNLKTHYAVHRSKGTTCHYCGKVFACTSALNIHYRSHTKERPFRCDCCSKGFSTKGNLKQHMLTHKIR
Gastropoda (Crepidula fornicata)	PNQCVICHRVLSCKSALQMHYRIHTGERPFKCKICNRKFTTKGNLKTHMGVHRTKSTTCPTCFKTFACRSALDIHMRSHTKERPFQCDECQRSFSTRGNLKQHQLTHRPD
Gastropoda (Lottia gigantea)	PNQCLICQRVLSCKSALQMHYRIHTGERPFKCKICSRSFTTKGNLKTHMGVHRAKPTTCNICFKTFACRSALDIHYRSHTKERPFSCDVCERTFTTKGNMRQHMLTHKIR
Gastropoda (Biomphalaria glabrata)	PNQCVVCHRVLSCKSALQMHYRIHTGERPFKCKICGRSFTTKGNLKTHMGVHRAKPTTCNICLKTFACKSALDIHYRSHSKLKPFQCEICERCFSTRGNLKQHLLTHKIR
Bivalvia (Crassostrea gigas)	PNQCVICHRVLSCKSALQMHYRIHTGERPYKCKICGRAFTTKGNLKTHMGVHRMKPTTCNICFKTFACRSALDIHYRSHTKERPYKCEVCDRSFTTKGNMKQHMLTHKIR
Cephalopoda (Octopus bimaculoides)	PNECAICHRVLSCKSALQMHYRIHTGERPFRCKLCGRAFTTKGNLKTHMGVHRAKPTTCYICFKTFACKSALDIHIRSHTKERPFKCEVCDRSFSTKGNMKQHMLTHKIR
Nemertea (Lineus ruber)	PNQCVICHRVLSCKSALQMHYRIHTGERPFRCKICGRSFTTKGNLKTHMGVHRAKPTTCNICTKVFACRSALDIHYRSHTKERPYMCEVCDRGFTTRGNMKQHMLTHKIR
Annelida (Dinophilus gyrociliatus)	PNQCAICERVLSCKSALQMHYRTHTGERPYRCKICGRRFTTKGNLKTHMGVHRGKPTTCNICYKTFACKSALDIHYRSHTKERPFQCDVCERSFTTKGNMKQHMLTHKIR
Brachiopoda (Terebratalia transversa)	PNQCVICHRVLSCKSALQMHYRIHTGERPFKCKICGRSFTTKGNLKTHMGVHRSKPTTCDICLKTFACLSALDIHYRSHTKEKPFVCQVCDRTFTTRGNMKQHMLTHKIR
Nemertodermatida (Meara stichopi)	PNQCHICHKVLSCRSALQMHYRIHTGEKPFECEVCGRCFTTKGNLKTHLVVHNNSPTSCEFCGKTFACQSALEIHLRCHTRERPFICHHCDRGFSTRGNLKQHLMTHEVS
Xenoturbellida (Xenoturbella bocki)	PNQCVICHRVLSCRSALQMHYRTHTGERPHKCKICGRAFTTKGNLKTHLSIHRTPPTACDVCGKAFACQSALNIHYRSHTKERPFRCHVCEKGFSTKGNLKQHMLTHKIR
Priapulida (Priapulus caudatus)	PNKCIVCNRVLSCRSSLNMHYRTHTGERPFKCRICSRGFTTKGNLKTHMGVHRIRLPTCRICFKQVHSHSELAIHMRVHGVDKSYRCEVCERSFLSRDNLKQHMLTHKIR
Crustacea (Limulus polyphemus)	PNQCVICHRVLSCRSALQMHYRTHTGERPFKCKICGRAFTTKGNLKTHMGVHRVKPTTCQICFKTFACQSALSIHYRSHTKERPFKCDACDRAFSTKGNMKQHMLTHKIK
Arachnida (Parasteatoda tepidariorum)	PNQCVFCHRVLSCKSALQMHYRTHTGERPFKCKICSRAFTTKGNLKTHMSVHRAKSTTCRICFKTFACNSALEIHYRSHTKERPFKCNVCDRGFTTKGNMKQHLLTHKSR
Lepidoptera (Papilio machaon)	PNECIVCHRVLSCKSALQMHYRTHTGERPFRCKLCGRAFTTKGNLKTHMGVHRIKPTTCNICYKTFACNSALEIHYRSHTKERPFKCTVCDRGFSTKSSGGG---CQCG-
Hymenoptera (Dufuorea novaeangliae)	PNQCVICHRVLSCKSALQMHYRTHTGERPFKCKICGRAFTTKGNLKTHMGVHRAKPTTCNICYKTFACNSALEIHYRSHTKERPFKCTICDRGFSTKNDGSGQQTCNCTS
Bryozoa (Membranipora membranacea)	PNQCLICKRPLSCKSALMMHYRTHTGERPFKCRLCNRAFTTKGNLKTHFGVHRAKPTTCDICGKQHGCRSALEIHYRSHTKHRPFKCEICGKAFTTRGNMKQHILTHKPE
