# Supplementary figures and images for: Molecular, phylogenetic and developmental analyses of Sall proteins in bilaterians
Source: EvoDevo. 2018 Apr 10;9:9. doi: 10.1186/s13227-018-0096-z (PMC5892016; doi:10.1186/s13227-018-0096-z)

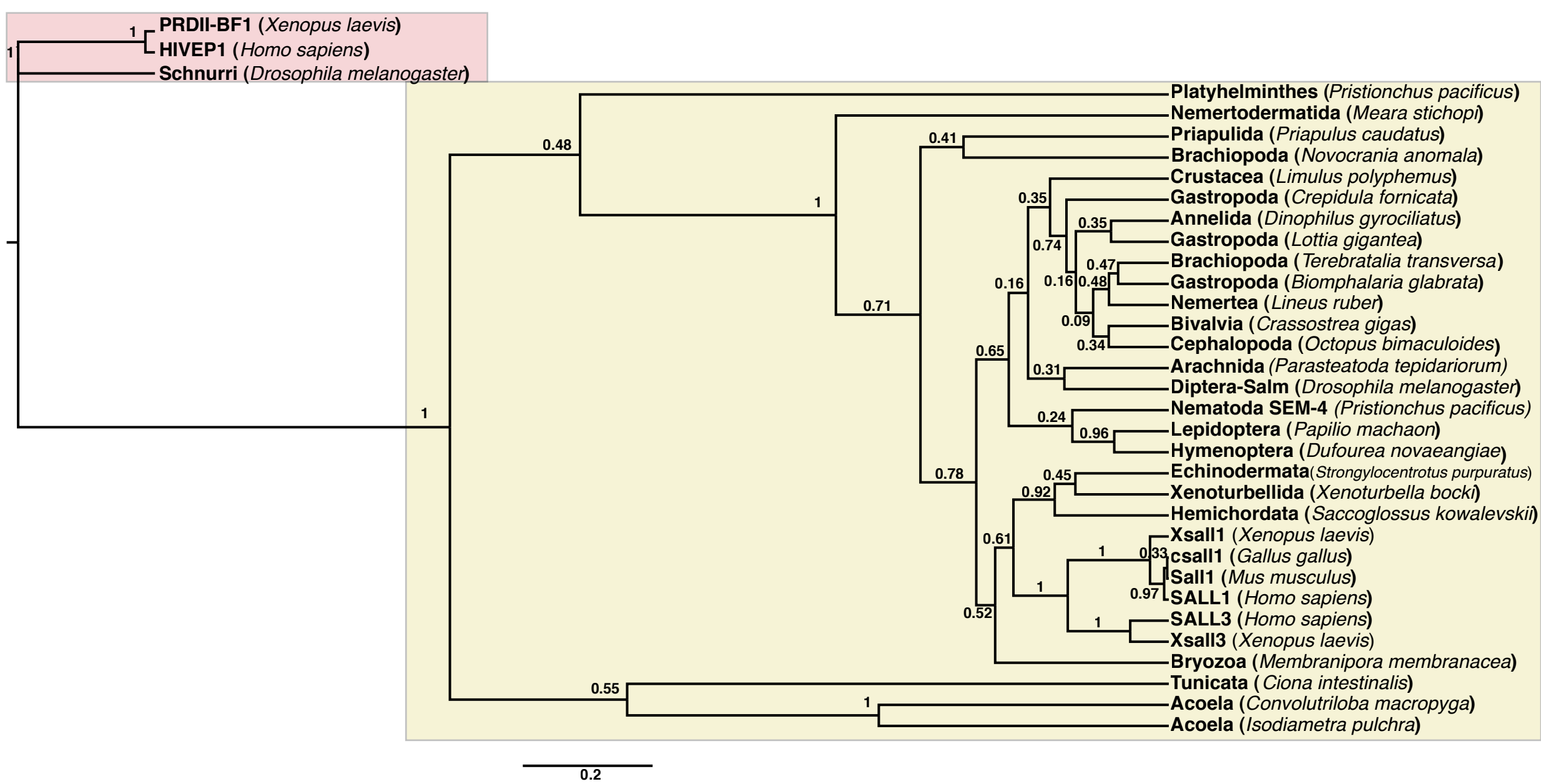

Supplement: Supplementary file 5 — Additional file 5: Fig. S3. Phylogenetic analysis of zinc-finger proteins containing the “Sal-box” (FTTKGNLK). Maximum clade credibility tree from the BEAST analysis including the deduced amino acid sequences of Sall proteins together with Schnurri, PRDII-BF1 and HIVEP1. Numbers indicated above the nodes are Bayesian posterior probabilities (BPP). The yellow box highlights Sall sequences across the Bilateria (BPP = 1) and the red box, the sequences of the other zinc-finger-related proteins (BPP = 1). [file 13227_2018_96_MOESM5_ESM.pdf]

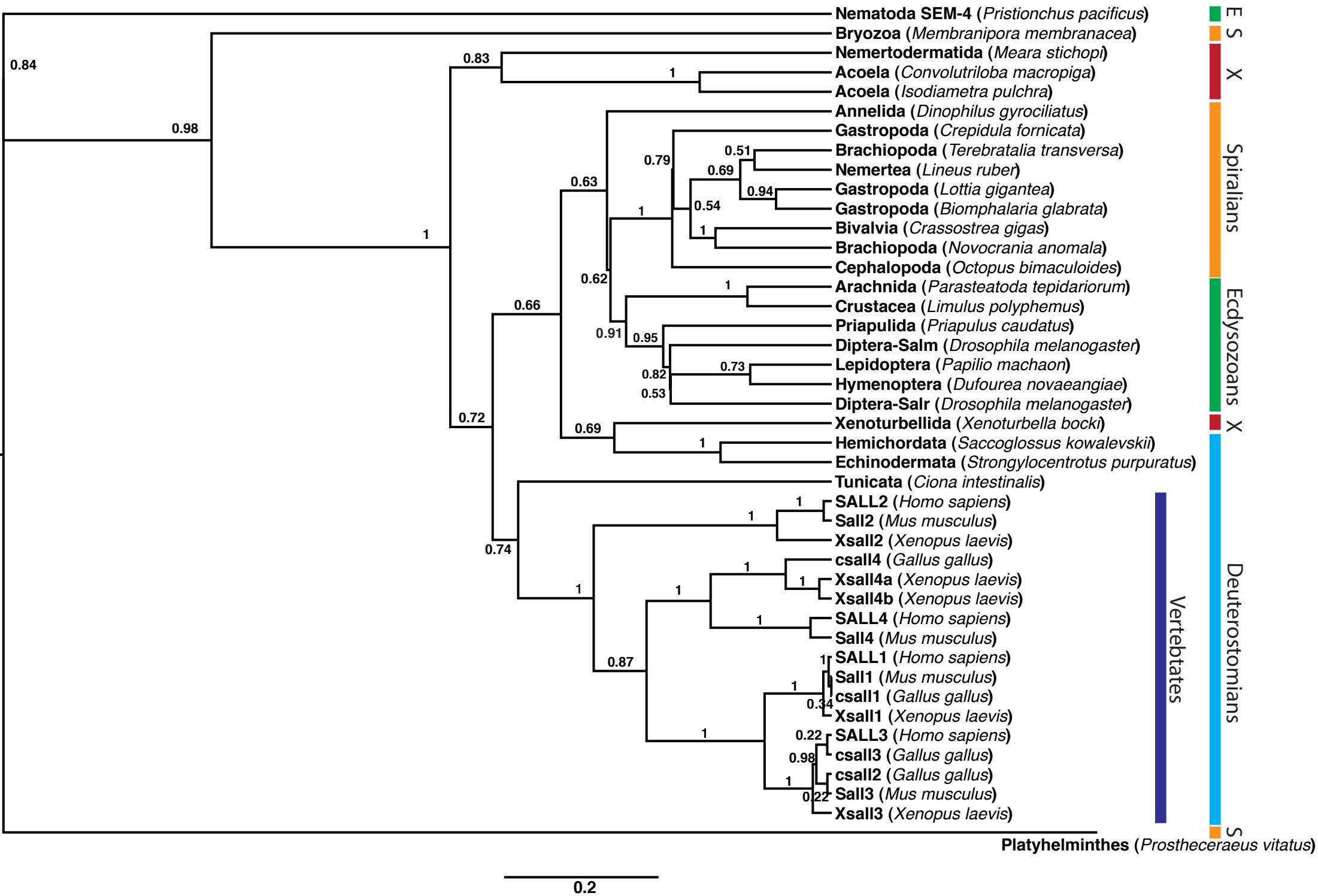

Supplement: Supplementary file 6 — Additional file 6: Fig. S4. Phylogenetic analysis of Sall proteins across the Bilateria. Maximum clade credibility tree from the BEAST analysis including the deduced amino acid sequences of Sall proteins. Numbers above nodes are Bayesian posterior probabilities. The dark blue line groups Sall sequences of Vertebrates; the light blue line, the sequences of deuterostomes; the green line, the sequences of ecdysozoans (E); the orange line, the sequences of spiralians (S); and the red line, the sequences of xenacoelomorphs (X). [file 13227_2018_96_MOESM6_ESM.pdf]

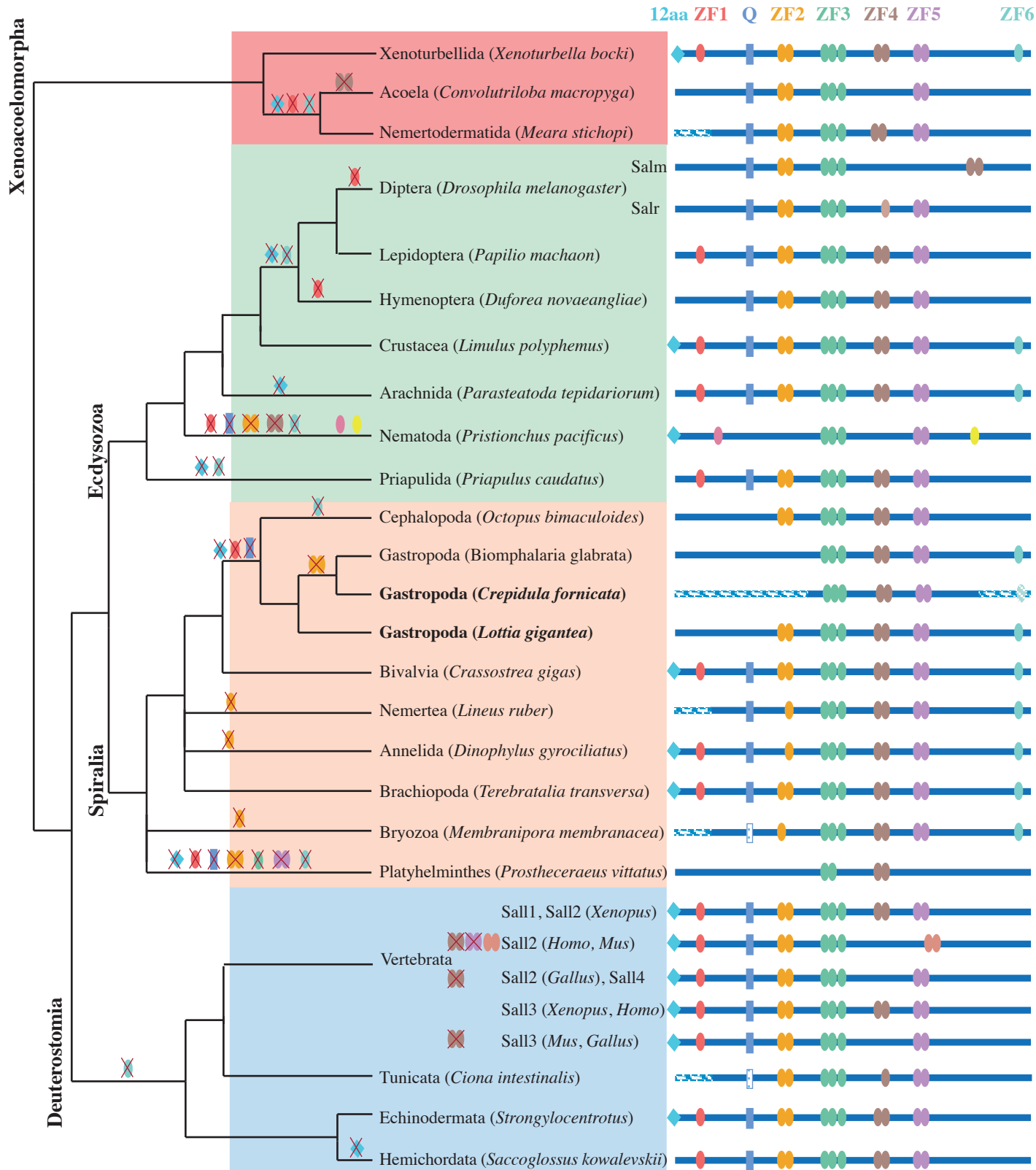

Supplement: Supplementary file 7 — Additional file 7: Fig. S5. Hypothesized Sall protein gains and losses during Bilateria evolution. The turquoise diamond represents the 12 conserved amino acids at the N-terminal end that interact with the HDC NuRD [14]. Colored ovals represent the zinc-finger motifs from ZF1 to ZF6. The blue rectangle represents the poly-Q region. The striped lines represent missing sequence. Phylogenetic tree based on Cannon et al. [63] and Peters et al. [64]. The blue box on the tree highlights the deuterostomes; the orange box, the spiralians; the green box, the ecdysozoans; and the red box, the xenacoelomorphs. Bold names highlight the snail species studied in more detail in this work. On each branch, proposed gains and losses of Sall protein domains are indicated. [file 13227_2018_96_MOESM7_ESM.pdf]

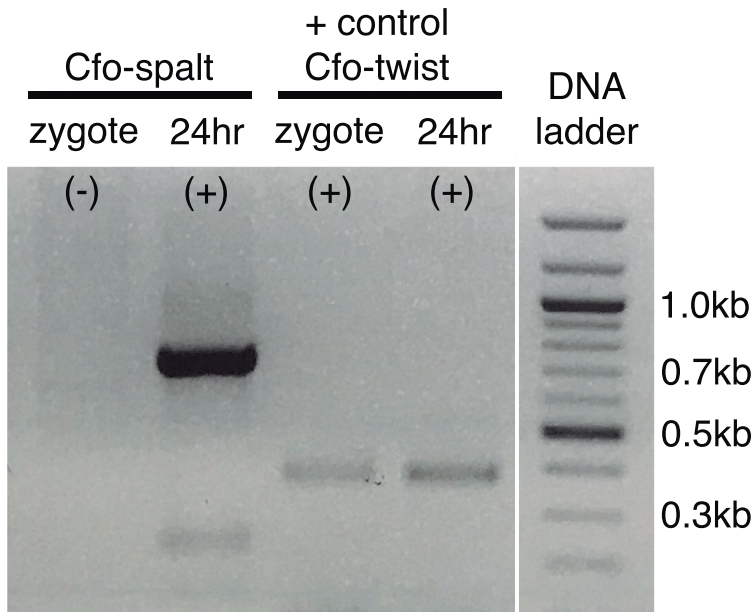

Supplement: Supplementary file 8 — Additional file 8: Fig. S6. Amplification of spalt from cDNA extracted from zygotes and 24-h post-fertilization Crepidula embryos. Amplification of spalt (Cfo-spalt) was only detected using cDNA from 24-h post-fertilization Crepidula embryos. There was no amplification at all when cDNA from zygotes was used. The gene twist from Crepidula (Cfo-twist) was used as a positive control since twist is detected at both stages of development. PCR products were run on 1% agarose gels. [file 13227_2018_96_MOESM8_ESM.pdf]

a

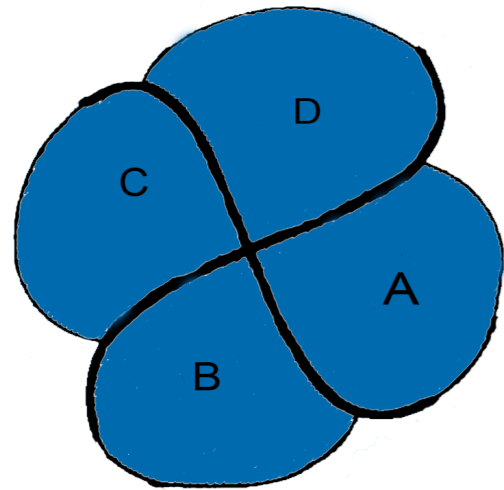

b

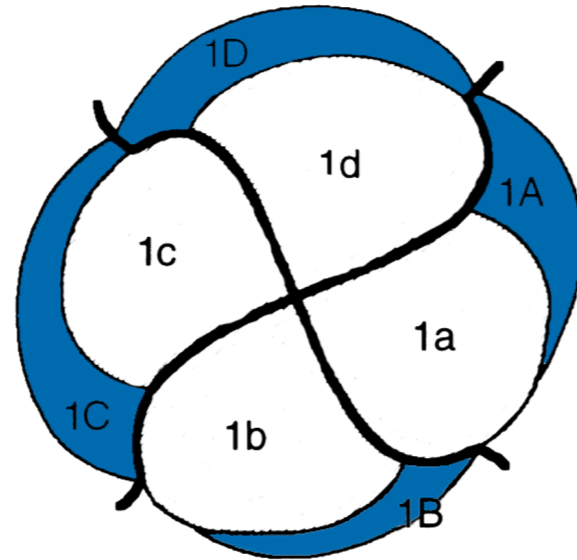

c

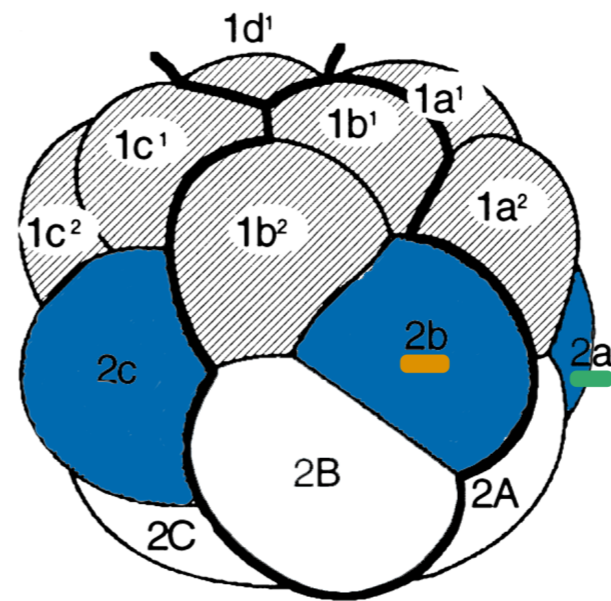

d

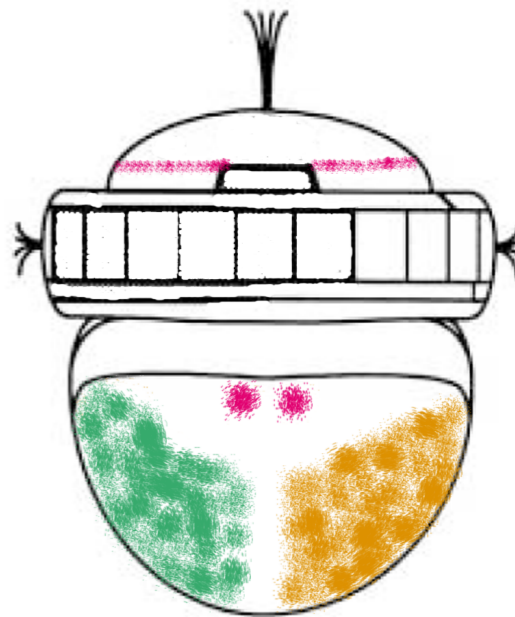

Supplement: Supplementary file 9 — Additional file 9: Fig. S7. Schematic drawings of sall expression at 4-cell, 8-cell, 16-cell stages and trochophore larva in L. gigantea. a: expression in all cells at 4-cell stage. b: Expression in the macromeres at 8-cell stage. c: Expression in the 2 m micromeres at 16-cell stage. Micromere 2a underlined in green and 2b underlined in orange. d: expression in the trochophore. In green and orange, expression of sall in the dorsolateral ectoderm derived from 2a and 2b, respectively. In pink sall, expression in the cephalic ring and next to the stomodeum, not derived from 2a and 2b micromeres. Drawings modified from Dictus and Damen [69]. [file 13227_2018_96_MOESM9_ESM.pdf]
