# Supplementary material for: Molecular, phylogenetic and developmental analyses of Sall proteins in bilaterians
Source: EvoDevo. 2018 Apr 10;9:9. doi: 10.1186/s13227-018-0096-z (PMC5892016; doi:10.1186/s13227-018-0096-z)
Supplement: Supplementary file 3 — Additional file 3: Fig. S2. Sequence alignment of Sal proteins. Alignment of the deduced amino acid sequences of Sall proteins of different taxa. Amino acids shared by more than 50% of the species are shaded in gray. [file 13227_2018_96_MOESM3_ESM.pdf]

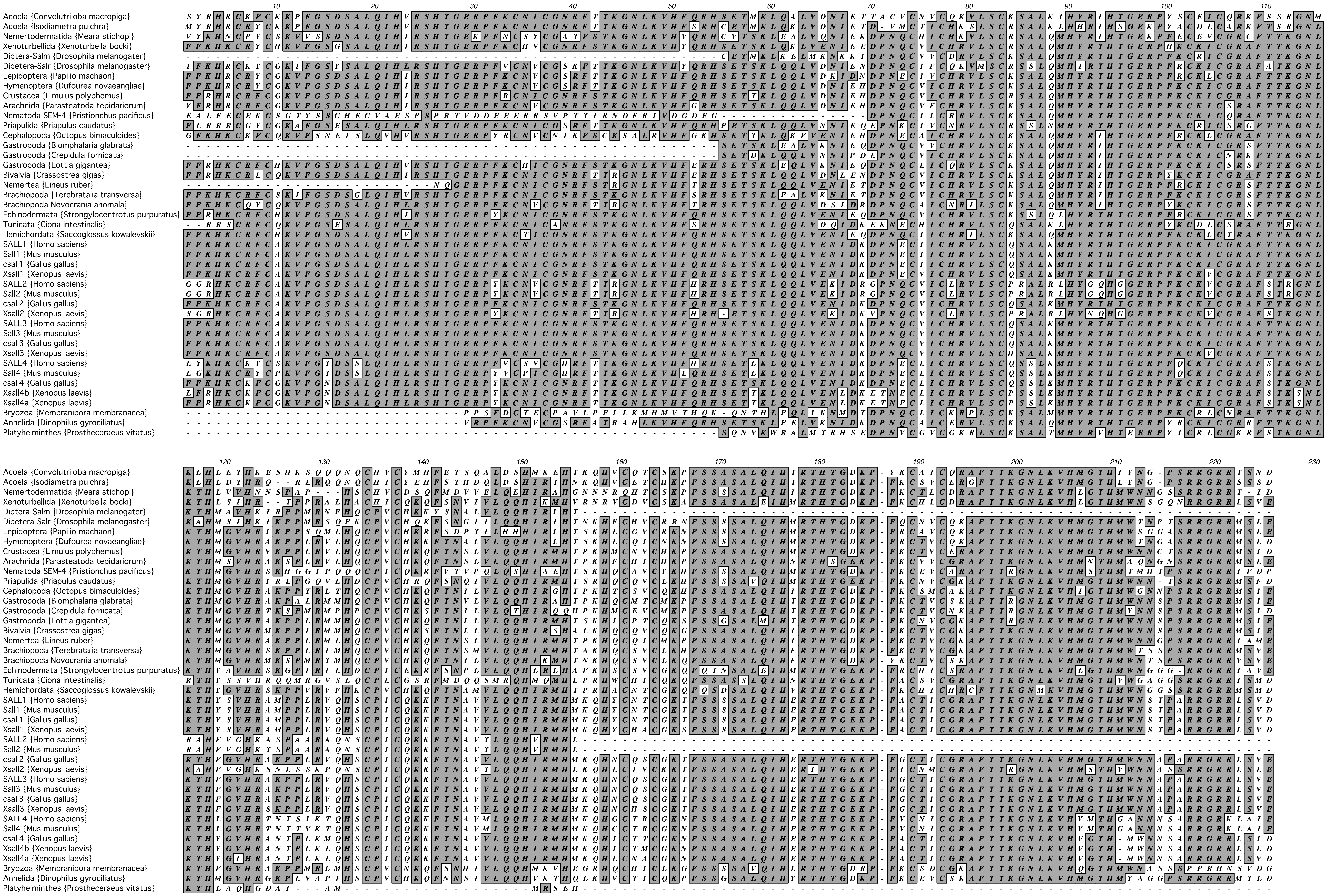
Acoela {Convolutriloba macropiga}
Acoela {Isodiametra pulchra}
Nemertodermatida {Meara stichopi}
Xenoturbellida {Xenoturbella bocki}
Diptera-Salm {Drosophila melanogater}
Dipetera-Salr {Drosophila melanogaster}
Lepidoptera {Papilio machaon}
Hymenoptera {Dufourea novaeangliae}
Crustacea {Limulus polyphemus}
Arachnida {Parasteatoda tepidariorum}
Nematoda SEM-4 {Pristionchus pacificus}
Priapulida {Priapulus caudatus}
Cephalopoda {Octopus bimaculoides}
Gastropoda {Biomphalaria glabrata}
Gastropoda {Crepidula fornicata}
Gastropoda {Lottia gigantea}
Bivalvia {Crassostrea gigas}
Nemertea {Lineus ruber}
Brachiopoda {Terebratalia transversa}
Brachiopoda Novocrania anomala}
Echinodermata {Strongylocentrotus purpuratus}
Tunicata {Ciona intestinalis}
Hemichordata {Saccoglossus kowalevskii}
SALL1 {Homo sapiens}
Sall1 {Mus musculus}
csall1 {Gallus gallus}
Xsall1 {Xenopus laevis}
SALL2 {Homo sapiens}
Sall2 {Mus musculus}
csall2 {Gallus gallus}
Xsall2 {Xenopus laevis}
SALL3 {Homo sapiens}
Sall3 {Mus musculus}
csall3 {Gallus gallus}
Xsall3 {Xenopus laevis}
SALL4 {Homo sapiens}
Sall4 {Mus musculus}
csall4 {Gallus gallus}
Xsall4b {Xenopus laevis}
Xsall4a {Xenopus laevis}
Bryozoa {Membranipora membranacea}
Annelida {Dinophilus gyrociliatus}
Platyhelminthes {Prostheceraeus vitatus}
Acoela {Convolutriloba macropiga}
Acoela {Isodiametra pulchra}
Nemertodermatida {Meara stichopi}
Xenoturbellida {Xenoturbella bocki}
Diptera-Salm {Drosophila melanogater}
Dipetera-Salr {Drosophila melanogaster}
Lepidoptera {Papilio machaon}
Hymenoptera {Dufourea novaeangliae}
Crustacea {Limulus polyphemus}
Arachnida {Parasteatoda tepidariorum}
Nematoda SEM-4 {Pristionchus pacificus}
Priapulida {Priapulus caudatus}
Cephalopoda {Octopus bimaculoides}
Gastropoda {Biomphalaria glabrata}
Gastropoda {Crepidula fornicata}
Gastropoda {Lottia gigantea}
Bivalvia {Crassostrea gigas}
Nemertea {Lineus ruber}
Brachiopoda {Terebratalia transversa}
Brachiopoda Novocrania anomala}
Echinodermata {Strongylocentrotus purpuratus}
Tunicata {Ciona intestinalis}
Hemichordata {Saccoglossus kowalevskii}
SALL1 {Homo sapiens}
Sall1 {Mus musculus}
csall1 {Gallus gallus}
Xsall1 {Xenopus laevis}
SALL2 {Homo sapiens}
Sall2 {Mus musculus}
csall2 {Gallus gallus}
Xsall2 {Xenopus laevis}
SALL3 {Homo sapiens}
Sall3 {Mus musculus}
csall3 {Gallus gallus}
Xsall3 {Xenopus laevis}
SALL4 {Homo sapiens}
Sall4 {Mus musculus}
csall4 {Gallus gallus}
Xsall4b {Xenopus laevis}
Xsall4a {Xenopus laevis}
Bryozoa {Membranipora membranacea}
Annelida {Dinophilus gyrociliatus}
Platyhelminthes {Prostheceraeus vitatus}
